# DNA methylation and DNA methyltransferases

**DOI:** 10.1186/s13072-017-0130-8

**Published:** 2017-05-08

**Authors:** John R. Edwards, Olya Yarychkivska, Mathieu Boulard, Timothy H. Bestor

**Affiliations:** 1 Center for Pharmacogenomics, Department of Medicine, Washington University School of Medicine, St. Louis, MO USA; 2 Department of Genetics and Development, College of Physicians and Surgeons of Columbia University, New York, NY USA

**Keywords:** Epigenetics, DNA cytosine methylation, Mammalian DNA methyltransferases, Methylation dynamics, Methylation-related human diseases

## Abstract

The prevailing views as to the form, function, and regulation of genomic methylation patterns have their origin many years in the past, at a time when the structure of the mammalian genome was only dimly perceived, when the number of protein-encoding mammalian genes was believed to be at least five times greater than the actual number, and when it was not understood that only ~10% of the genome is under selective pressure and likely to have biological function. We use more recent findings from genome biology and whole-genome methylation profiling to provide a reappraisal of the shape of genomic methylation patterns and the nature of the changes that they undergo during gametogenesis and early development. We observe that the sequences that undergo deep changes in methylation status during early development are largely sequences without regulatory function. We also discuss recent findings that begin to explain the remarkable fidelity of maintenance methylation. Rather than a general overview of DNA methylation in mammals (which has been the subject of many reviews), we present a new analysis of the distribution of methylated CpG dinucleotides across the multiple sequence compartments that make up the mammalian genome, and we offer an updated interpretation of the nature of the changes in methylation patterns that occur in germ cells and early embryos. We discuss the cues that might designate specific sequences for demethylation or de novo methylation during development, and we summarize recent findings on mechanisms that maintain methylation patterns in mammalian genomes. We also describe the several human disorders, each very different from the other, that are caused by mutations in DNA methyltransferase genes.

## The shape of genomic methylation patterns

The current human genome assembly contains ~3 × 10^7^ CpG dinucleotides, each of which can exist in the methylated or unmethylated state. The number of possible methylation patterns in a single haploid genome far exceeds the number of atoms in the observable universe; this greatly increases both the potential information content of the genome and the difficulty of statistical analysis [1, 2].

**Fig. 1:**
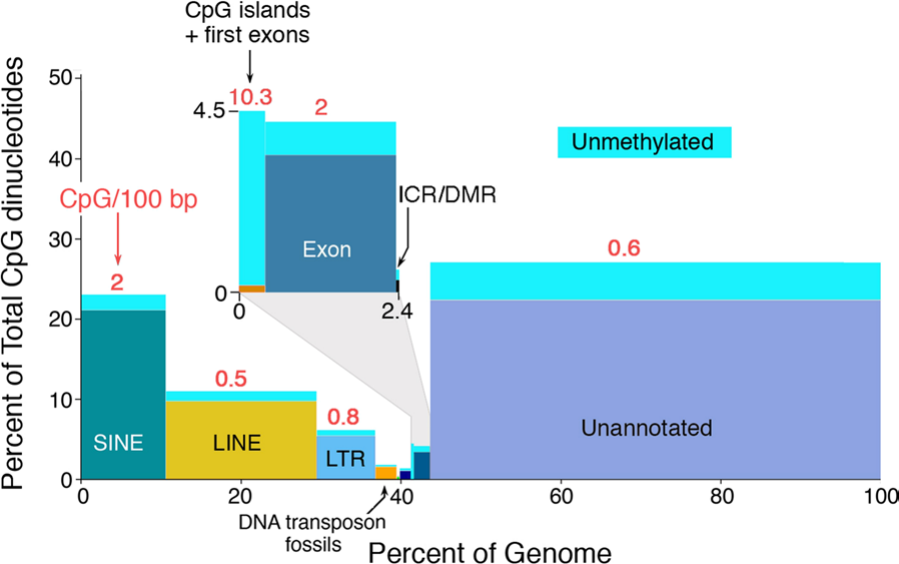
Distribution of DNA methylation across sequence compartments in the human genome. *Vertical axis* indicates percentage of total CpG dinucleotides in each indicated compartment; *horizontal axis* indicates percentage of total genome in each compartment; *light blue* at the *top* of each compartment indicates unmethylated fraction. Numerals in *red* denote CpG dinucleotides per 100 bp. The genome-wide CpG density expected on the basis of G + C content is 4.2 per 100 bp. Note that the only sequence compartment that exists in the largely unmethylated state is the CpG island/first exon compartment; this compartment occupies <0.5% of the genome. The ICR/DMR compartment (differentially methylated regions of imprinting control regions) represents ~0.001% of the genome and ~0.01% of total CpG dinucleotides. Introns are included in the unannotated compartment, as are putative enhancers. The methylation data are from Bisulfite-Seq data for hippocampus (Roadmap Epigenome Project sample E071 [5]), but other differentiated adult tissues show very similar trends

Whole-genome methylation profiling has recently made it possible to assign approximate methylation levels to the multiple sequence compartments that make up the human genome [1–3]. We have analyzed this compartment-specific methylation as it occurs in differentiated somatic cells; the data are shown in Fig. 1. Transposon-derived sequences (SINE, LINE, and LTR) are abundant and densely methylated; the remainder of the genome is more variably methylated, with promoter-associated CpG islands and first exons representing the only sequence compartment that is largely unmethylated. Seventy-five percent of all promoters are within CpG islands and unmethylated [1–4]; the remaining promoters have very low CpG densities, and methylation is very unlikely to regulate their expression [1, 2]. Many CpG islands are not associated with promoters or other annotated regulatory sequences, and their methylation status is of unknown and possibly inconsequential biological significance.

Current evidence indicates that the primary biological functions of DNA methylation lie in the heritable transcriptional repression of retrotransposons, the monoallelic expression of imprinted genes, X chromosome inactivation in female cells, and the selective exposure of promoters of cellular genes to transcription factors. There is evidence that genomic methylation patterns at regulatory sequences are essentially static during development, although CpG-poor promoters can show partial demethylation upon transcriptional activation that is likely to be a consequence rather than a cause of activation [2].

Multiple lines of evidence indicate that only ~10% of the mammalian genome is functional, as shown by comparative biology studies and by the fact that most of the genome is evolving at the neutral rate and does not appear to be under selection [6, 7]. Most DNA methylation is also likely to be without significant biological function; this is consistent with high rate of loss of CpG dinucleotides across most of the genome during evolution [8] and the highly heterogeneous nature of genomic methylation patterns even in single tissue types [1, 3].

## Methylation dynamics during development

**Fig. 2:**
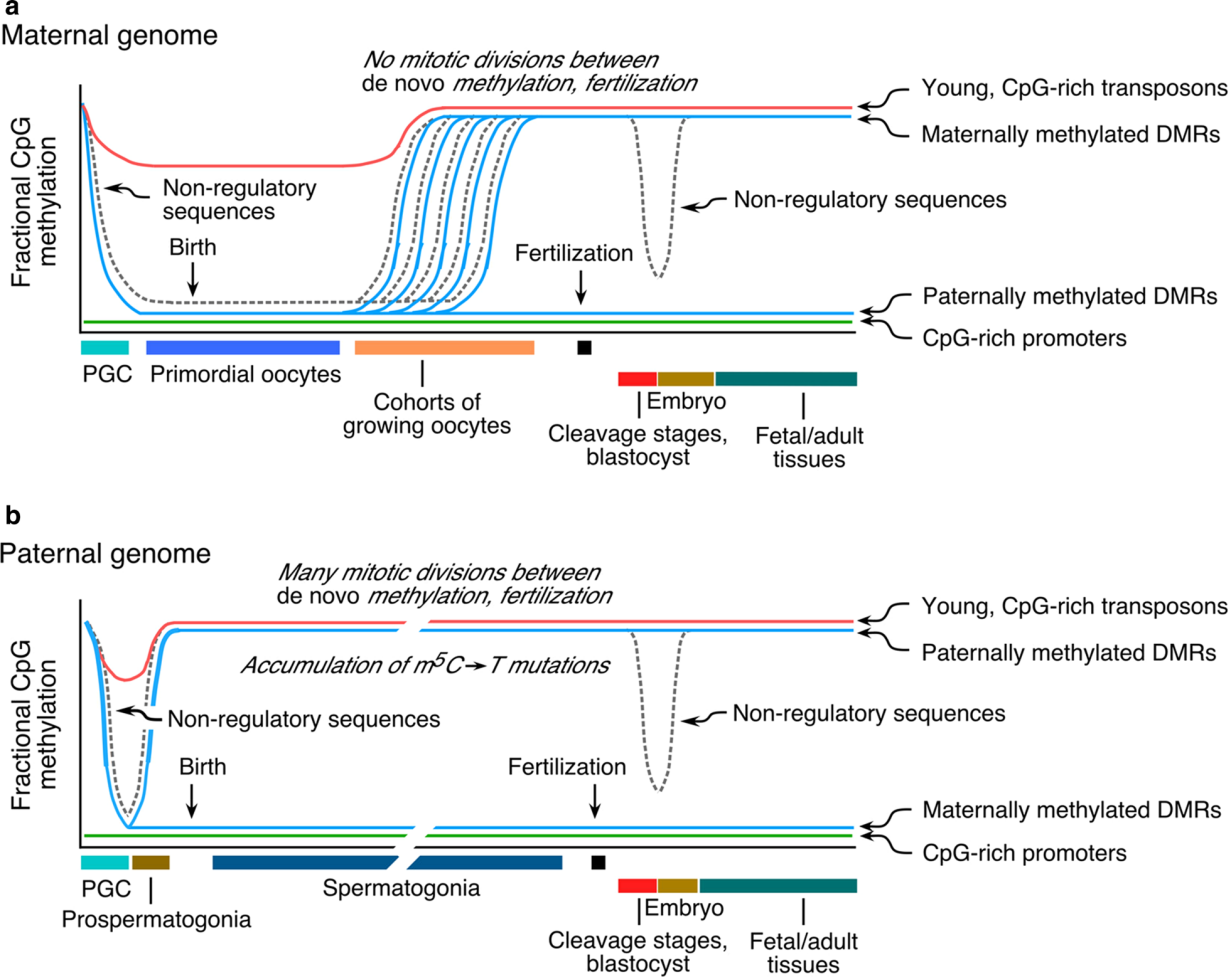
Dynamics of demethylation and de novo methylation in the maternal (**a**) and paternal (**b**) genomes during mammalian development. The standard depictions of developmental changes in genomic methylation patterns often assume a monolithic genome; in fact, different sequence compartments display marked differences in timing of methylation and demethylation. CpG-rich (CpG island) promoters are unmethylated at all stages, except for the small number of CpG islands associated with imprinting control regions and CpG islands on the inactive X chromosome in somatic cells of females. Young, CpG-rich transposons largely escape both waves of demethylation. Most of the dynamic methylation and demethylation that occurs in primordial germ cells (PGCs) and the early embryo affects sequences that are evolving at the neutral rate and whose methylation status is without known biological effect. The methylation status of these sequences, which represent the bulk of the genome and are composed of satellite DNA, old and inactive transposons, introns, and unannotated sequences evolving at the neutral rate, is shown by *broken lines*

Since 1987, it has been held that there are two waves of demethylation and remethylation [9] during development that in the standard depictions are implied to affect virtually the entire genome (reviewed in [10]). Under this model, the first wave of demethylation occurs during the migration of proliferating primordial germ cells, with remethylation occurring in postmigratory germ cells; the second wave of demethylation takes place in cleavage stage embryos and results in a minimum in DNA methylation at the blastocyst stage. As shown in Fig. 2, this standard double-dip model obscures the methylation dynamics of the small fraction of the genome shown in Fig. 1 where methylation is likely to exert regulatory effects. First, the large majority of CpG island promoters are not subject to these waves of methylation and demethylation because they are unmethylated at all stages. Second, the methylation status of alleles at differentially methylated regions (DMRs) of imprinting control regions (ICRs) changes at different developmental stages: they are demethylated in primordial germ cells and remethylated in cohorts of growing oocytes shortly before ovulation [11] and in the entire population of prospermatogonia around the time of birth [12]. The sex-specific methylation at ICRs/DMRs escapes the demethylation that occurs in cleavage stage embryos. Third, the small population of young, CpG-rich transposons largely escapes demethylation both in primordial germ cells [13] and in the early embryo [14]. The types of sequences that undergo the double wave of demethylation and remethylation are largely composed of old and inactive transposon remnants, satellite and other repeated DNA, and the unannotated and rapidly diverging fraction of the genome that shows little evidence of biological function. Figure 2 shows that the dynamics of demethylation and remethylation during development are more complex than depicted in the double-dip model and that sequences whose methylation status is of biological importance do not conform to this model.

Figure 2 also shows the basis for the pronounced sexual dimorphism in the rate of C → T mutations driven by deamination of 5 methylcytosine (m^5^C), which converts the base directly to T. De novo methylation of DMRs/ICRs and most of the genome occurs in the entire population of male germ cells around the time of birth; these methylation patterns exist for the reproductive life of the organism and must be propagated by maintenance methylation in spermatogonia through many mitotic divisions prior to entry into meiosis [12]. In female germ cells, de novo methylation takes place in growing oocytes, which are arrested in meiosis I and undergo no mitotic divisions prior to fertilization [11]; there is therefore very little opportunity for deamination of m^5^C to occur. As a result of this sexual dimorphism, de novo mutations at CpG dinucleotides are much more common in spermatozoa [15]; many sporadic genetic disorders are caused primarily by C → T mutations at methylated CpG dinucleotides at alleles of paternal origin. Furthermore, paternally methylated ICRs/DMRs have been eroded by C → T mutations over evolutionary time and are far fewer in number and have become reduced in CpG density as compared to maternally methylated ICRs/DMRs [16].

## Attracting and repelling DNA methylation

The cues that designate specific sequences for de novo methylation, faithful versus error-prone maintenance methylation, or demethylation at different developmental stages are not well understood. It is clear that the default state of most of the genome is partially to densely methylated [1, 3]. This is shown by the fact that removal of most DNA methylation in somatic cells by treatment with DNA methyltransferase inhibitors is followed by gradual remethylation of most sequences after withdrawal of the inhibitor [17]; this methylation occurs largely at sequences that are unlikely to have appreciable biological effects. Restoration of DNMT1 to *Dnmt1*-null ES cells, whose genomes have lost nearly all m^5^C, also results in the remethylation of most of the genome, but with a failure to reestablish methylation at imprinting control regions until these sequences have been passed through the germ line [18].

Repeated sequences can attract de novo methylation; a transgene array of tandem repeats became methylated in transgenic mice, but methylation was lost when the repeat array was reduced to a single unit [19]. Other mechanisms by which repeated sequences might be targeted for de novo methylation have been discussed [20], although the actual mechanism by which repeated sequences attract de novo methylation has not been defined.

The mechanisms that designate specific sequences for de novo methylation in the germ line are only partially understood. Deletion of the gene that encodes DNMT3L (which is related to DNMT3A and DNMT3B in framework regions but lacks the domains involved in transmethylation) causes a failure of de novo methylation in prospermatogonia [12] and in growing oocytes [11], the only cell types in which DNMT3L is expressed. DNMT3L forms a complex with DNMT3A and DNMT3B, and DNMT3L targets this complex to DNA sequences associated with histones that are unmethylated at lysine 4 of histone H3 (H3K4); unmethylated H3K4 is associated with inactive promoters and with methylated DNA [21]. Ablation of the Argonaute proteins MILI or MIWI2, which are expressed in early germ cells and are involved in the biogenesis of PIWI-interacting RNAs (piRNAs), causes a failure of de novo methylation very similar to that seen in *Dnmt3L*-null germ cells, although DNA methylation is affected only in male germ cells [22]. This finding implies that piRNAs are upstream of histone H3K4 methylation and demethylation, which in turn are upstream of the DNMT3L/DNMT3A/DNMT3B complex. However, it is not known how piRNAs affect H3K4 methylation and no connection between piRNAs and the DNMT3L/DNMT3A/DNMT3B complex has been identified. DNMT3A and DNMT3B have also been shown to bind to H3K36me3 through their PWWP domains [23].

The binding of transcription factors to promoters even in the absence of active transcription can cause the loss of DNA methylation in the vicinity of the binding site [24]; even the binding of lac repressor can cause the loss of DNA methylation from CpG sites near *lac* operators in transfected mammalian cells [25]. Many of the expression–methylation correlations that have been reported since 1978 [26] are likely to be a consequence of transcriptional activation rather than a cause [2]. These effects are largely restricted to sequences of low CpG density. The expression of the large majority of genes does not markedly change after global genome demethylation [2].

CpG island promoters are protected from de novo methylation at essentially all developmental stages. Exceptions are a small number of promoters at ICR/DMRs [27] and CpG island promoters on the inactive X chromosome in female somatic cells [28]. The mechanism that protects CpG island promoters does not involve sequestration of the promoters in condensed chromatin since unmethylated CpG-rich sequences in nuclei show the greatest accessibility to diffusible factors such as DNase I [1].

Although the mechanisms that protect most CpG island promoters from de novo methylation are not understood, a specific class of CpG island promoters is protected from de novo methylation by the multidomain chromosomal protein FBXL10 (also known as KDM2B, JHDM1B, and CXXC2); these are the CpG island promoters bound by polycomb repressive complexes (PRC) 1 and 2. In the absence of FBXL10, PRC-bound promoters undergo de novo methylation with concomitant silencing of gene expression [29]. Even though FBXL10 is bound to essentially all CpG island promoters, removal of FBXL10 induces de novo methylation and transcriptional silencing only of that small subset of CpG island promoters that are bound both by FBXL10 and by PRC 1 and 2. This implies that PRC 1 and/or 2 has a tendency to attract de novo methylation and that FBXL10 has evolved to counteract this activity. Inhibition of de novo methylation is likely to involve the CXXC domain of FBXL10; this domain, which is found in ~14 nuclear proteins, binds specifically to unmethylated CpG dinucleotides [30]. The methylation abnormalities that arise in cells that lack FBXL10 are strikingly similar to those seen in pediatric ependymomas and some other pediatric cancers; the methylation abnormalities appear to be important drivers of tumorigenesis as very few mutations have been detected in these tumors [31]. The processes that render PRC-bound promoters subject to de novo methylation in these tumors are not currently understood.

## Mechanisms that mediate faithful maintenance methylation by DNMT1

That genomic methylation patterns are subject to mitotic inheritance in somatic cells was predicted to occur in 1975 [32, 33] and demonstrated experimentally in 1981 [34]. Maintenance methylation can be very faithful; allele-specific methylation patterns established at ICRs/DMRs in germ cells of the preceding generation can be maintained in offspring through to adulthood with little alteration, and the two X chromosomes in female cells maintain different methylation patterns from soon after implantation of the embryo to the end of life.

However, maintenance methylation is less efficient at other sequences; methylation patterns can be heterogeneous in single tissue types [1, 3] and even in clonal cell populations. Heterogeneous methylation is observed largely at sequences without discernable regulatory activity. DNMT1 has long been known to preferentially methylate hemimethylated DNA (the product of semiconservative DNA replication; reviewed in [35]), but only recently have structural studies begun to reveal the mechanism.

**Fig. 3:**
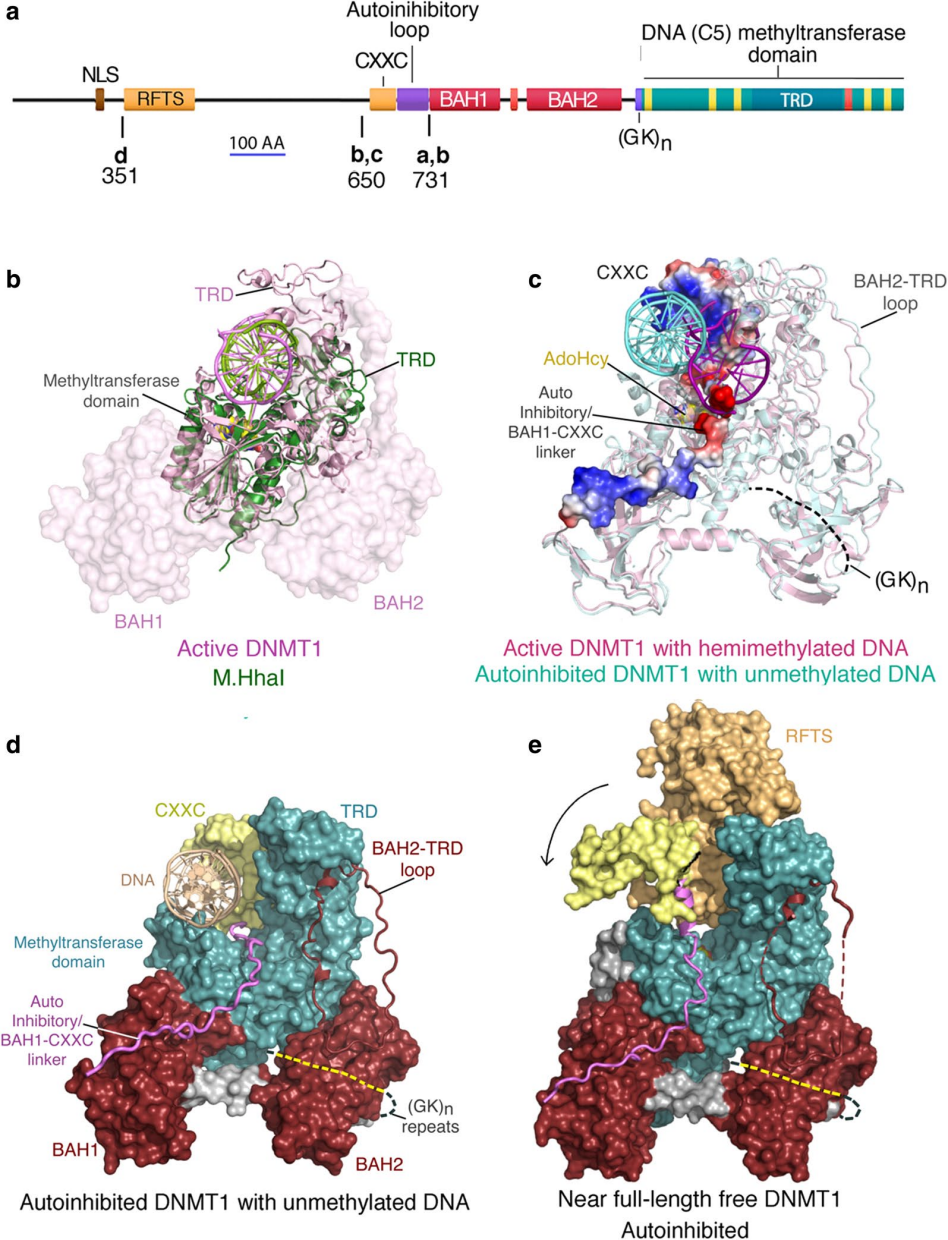
Structure and regulation of DNMT1. **a** Functional domains in DNMT1. A nuclear localization sequence (NLS) and replication focus targeting sequence (RFTS) are closest to the N-terminus. A CXXC domain binds selectively to unmethylated CpG dinucleotides; this binding event interposes an acidic autoinhibitory loop between the active site and unmethylated DNA to inhibit de novo methylation [30]. The bromo-adjacent homology (BAH) domains 1 and 2 are of unknown function but are related in structure to BAH domains in other proteins that bind to specific modified histones (reviewed in [39]). A run of alternating lysine and glycine residues joins the multidomain N-terminal domain to the large C-terminal methyltransferase domain, which is related in sequence and structure to all other DNA (cytosine-5) methyltransferases (reviewed in [35]). *Letters* below the diagram indicate the position of N-terminal truncations in the crystal structures shown in **b**–**e**. **b** Superposition of the structures of active DNMT1 [30] and M.*Hha*I, a bacterial restriction methyltransferase [40]. The methyltransferase domain of DNMT1 shows strong isostery with full-length M.*Hha*I. **c** Superposition of autoinhibited DNMT1 in complex with unmethylated DNA and active DNMT1 deleted for the CXXC and autoinhibitory loop domains in complex with hemimethylated DNA [41]. DNA can be seen to have accessed the catalytic pocket of DNMT1 in the active complex and to be very close to the *S*-adenosyl-l-homocysteine present in both complexes. **d**, **e** Impingement of the RFTS on the CXXC domain displaces the latter (*curved arrow*) into a conformation that inhibits binding of DNA [42]. It is proposed that the interaction of UHRF1 bound to hemimethylated DNA causes a retraction of the RFTS domain to allow access of hemimethylated DNA to the active site of DNMT1 [42]

As shown in Fig. 3, DNMT1 has a C-terminal catalytic domain related in sequence and structure to other DNA (cytosine-5) methyltransferases (including bacterial restriction methyltransferases such as M.*Hha*I) and a large N-terminal region that contains multiple functional domains. The CXXC domain (which is closely related to that of FBXL10) binds to unmethylated CpG dinucleotides and interposes a stretch of highly acidic amino acids (the autoinhibitory BAH1-CXXC linker in Fig. 3c and d) between the DNA and the active site of DNMT1, thereby inhibiting de novo methylation [30]. This autoinhibitory mechanism provides a several-fold preference of DNMT1 for hemimethylated DNA, but this is not sufficient to explain the faithfulness of in vivo maintenance methylation. A second mechanism that increases the preference of DNMT1 for hemimethylated DNA involves the interaction of the replication focus targeting sequence (RFTS) of DNMT1 with the multidomain protein UHRF1 (ubiquitin-like with PHD and ring finger domains 1), which contains an SRA domain that binds to hemimethylated CpG dinucleotides [36]. In the free protein, the RFTS of DNMT1 occludes access of DNA to the active site by impingement on the CXXC domain; it is proposed that the UHRF1/hemimethylated DNA complex displaces the inhibitory RFTS domain of DNMT1 in a handoff reaction to transfer hemimethylated DNA from UHRF1 to the active site of DNMT1. This proposal is consistent with the finding that UHRF1 is required for maintenance methylation in vivo; null alleles of *Uhrf1* phenocopy null alleles of *Dnmt1* in mice [36]. UHRF1 has multiple additional functional domains (including a tandem tudor domain that has been reported to bind to methylated H3K9 and to regulate the fidelity of maintenance methylation [37]), but mutation of UHRF1 so as to eliminate H3K9 binding had little effect on maintenance methylation in vivo [38].

DNMT1 also contains two bromo-adjacent homology (BAH) domains that occur in a number of other proteins, where some have been shown to bind to specific modified histones (reviewed in [39]). The function of the BAH domains in DNMT1 is unknown, although they may increase the efficiency of maintenance methylation by interaction with unidentified histones or histone modifications. While purified DNMT1 does have a modest intrinsic preference for hemimethylated DNA in vitro, it has recently become clear that multiple additional regulatory inputs, especially those mediated by the interaction with UHRF1, are required in vivo to ensure stable maintenance methylation at ICR/DMRs and other sequences where DNA methylation is subject to stable somatic inheritance.

## Pathogenic mutations in DNA methyltransferase genes

**Fig. 4:**
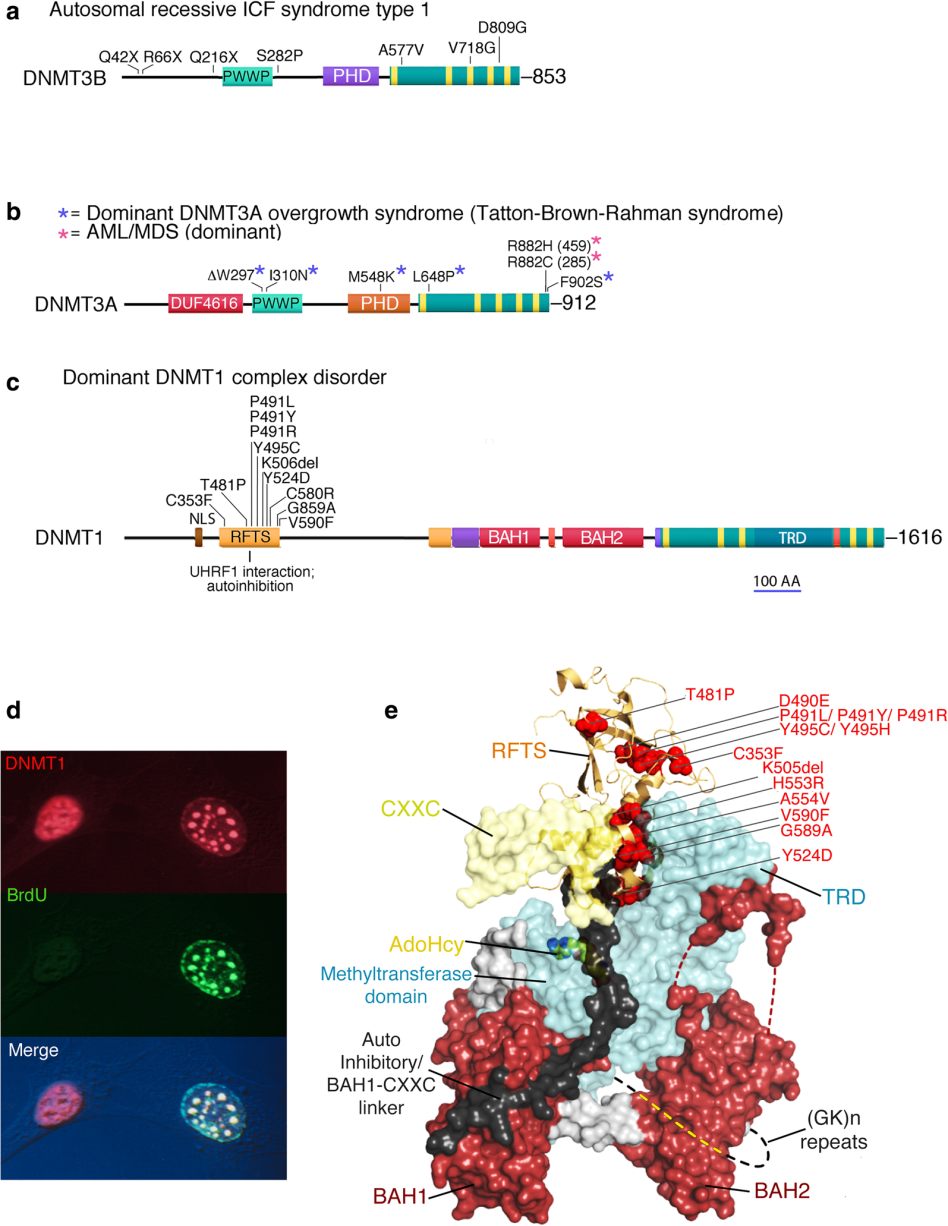
Each of the three DNMT genes is mutated in specific and diverse human syndromes. **a** DNMT3B bears recessive loss-of-function mutations in ICF syndrome type 1. **b** DNMT3A is mutated in dominant DNMT3A overgrowth syndrome and in subset of cases of acute myeloid leukemia and myelodysplastic syndrome. While most AML/MDS mutations affect codon 882, mutations at other positions also occur. **c** The RFTS domain of DNMT1 is subject to many different dominant mutations in a variable adult-onset cerebellar ataxia, deafness, dementia, and narcolepsy syndrome. The RFTS mediates interactions with replication foci during S phase (**d**) and with UHRF1. The positions of the amino acid substitutions within the structure of DNMT1 are shown in **e**. Only a subset of reported disease-associated mutations are shown for any of the three genes

All three human genes that encode active DNA methyltransferases have been found to be mutated in specific human diseases, although gross methylation abnormalities have been observed only in ICF syndrome type 1 (immunodeficiency, centromere instability, facial anomalies; OMIM 602900), which is caused by homozygous loss-of-function mutations at *DNMT3B* ([43]; Fig. 4a). ICF syndrome type 1 patients present with a variable combined immunodeficiency that is usually fatal prior to adulthood, mild but stereotypical facial abnormalities, and severe instability of classical satellite DNA on chromosomes 1, 9, and 16 that leads to gains and losses of chromosome arms to produce multiradiate or pinwheel chromosomes in phytohaemagglutinin (PHA)-stimulated T cells. Classical satellite DNA is almost completely unmethylated in all cells of patients with this syndrome, but chromosome instability is apparent only in certain cell types. Variable losses of methylation in other regions of the genome have also been reported [44], but the methylation abnormalities responsible for the pathogenesis of ICF syndrome cannot be specified with any confidence. While point mutations in *DNMT3B* in ICF syndrome type 1 can eliminate all enzyme activity [43], ICF patients homozygous for deletion or early truncation alleles have not been reported. Null alleles of *Dnmt3B* in mice are embryonic lethals [45], which suggests that DNMT3B protein, even if enzymatically inactive, is required for proper assembly and function of a complex that contains other factors.

Heterozygous somatic mutations in *DNMT3A* are present in ~15% of cases of acute myeloid leukemia (AML; OMIM 601626) [46] and in a smaller percentage of cases of myelodysplastic syndrome. Most mutations affect a single codon: R882 (encoded by CGC). C → T mutations at a methylated CpG dinucleotide within this codon convert it to a cysteine codon (TGC) if the top strand is mutated and to a histidine codon (CAC) if the bottom strand is mutated (Fig. 4b). It is not known whether methylation abnormalities are involved in those AML cases that bear mutations in DNMT3A. Mutations in DNMT3A are uncommon in neoplastic conditions other than certain leukemias and myelodysplastic syndrome.

Tatton–Brown–Rahman syndrome (OMIM 602769) is an overgrowth syndrome that involves tall stature, characteristic facial anomalies, and variable intellectual disabilities. The original authors referred to this condition as DNMT3A overgrowth syndrome [47]. Patients are heterozygous for germ line mutations in *DNMT3A* different from those reported to occur somatically in AML (Fig. 4b). It is not known whether DNA methylation abnormalities are present in Tatton–Brown–Rahman syndrome; the early-onset overgrowth phenotype is not inconsistent with defects in imprinted gene expression. Although there are no methylation data on this point, there are strong phenotypic similarities among Tatton–Brown–Rahman syndrome, Weaver syndrome (associated with heterozygous missense mutations in *EZH2*; OMIM 277590), and Sotos syndrome (usually associated with heterozygous loss-of-function mutations in *NSD1*; OMIM 117550) and the imprinting disorder Beckwith–Wiedemann syndrome (OMIM 130650), which strengthens the possibility that all these syndromes involve disruption of normal imprinted gene expression (reviewed in [47]).

Multiple dominant germ line mutations clustered in a single small domain of DNMT1 cause a heterogeneous group of adult-onset neurological disorders that include ataxia, sensorineural deafness, narcolepsy, dementia, psychosis, and other neurological and psychiatric abnormalities (OMIM 126375 and 605712) that are collectively known as autosomal dominant DNMT1 complex disorder [48]. All the known causative mutations involve single amino acid substitutions within the replication focus targeting sequence (RFTS; Fig. 4c), which mediates the interaction of DNMT1 and UHRF1 and the recruitment of DNMT1 into replication foci during S phase; in non-S phase cells, DNMT1 has a diffuse nucleoplasmic distribution (Fig. 4d). The locations of the causative mutations within the structure of DNMT1 are shown in Fig. 4e.

That mutations in *DNMT1* should cause adult-onset neurological defects without involvement of other tissues is unexpected; partial loss-of-function alleles of *Dnmt1* in mice cause pervasive developmental delays and high rates of leukemia without obvious neurological abnormalities [49]. Furthermore, adult neurons are postmitotic and perform little or no maintenance methylation. DNMT1 protein is nonetheless present at appreciable levels in neurons, and the mutated proteins show a tendency to form cytoplasmic aggregates when overexpressed in cultured cells [48]. It is likely that the toxicity of these aggregates (if they form in neurons of affected individuals), rather than an effect on DNA methylation, underlies the neuropathies caused by mutations in *DNMT1.* This interpretation is consistent with the report of a lack of obvious phenotypes after conditional deletion of the *Dnmt1* gene in postmitotic neurons of mice [50].

DNMT3L, which is expressed only in prospermatogonia and in growing oocytes, recruits DNMT3A and DNMT3B to nucleosomes that contain unmethylated H3K4 [51]. No disease-associated mutations in the *DNMT3L* gene have been reported in humans; based on mouse models, homozygous null alleles would be expected to produce non-syndromic azoospermia in males and maternal-effect embryonic lethality in the offspring of homozygous mutant females and normal males [11, 12].

The human biology of DNA methyltransferases illustrates the complex and enigmatic effects of disturbances of genomic methylation patterns on phenotype. The four human disorders firmly associated with mutations in DNA methyltransferase genes have largely non-overlapping phenotypes: one is germ line, recessive, early onset and involves a usually severe combined immunodeficiency (ICF syndrome type 1), one is germ line, dominant, adult onset and progressive and affects the central nervous system (DNMT1 complex disorder), another is germ line, dominant, early onset and involves overgrowth and intellectual disabilities without pronounced neurological disturbance (Tatton–Brown–Rahman or dominant DNMT3A overgrowth syndrome), and another is somatic, dominant and is involved in the etiology of lymphoid neoplasms (*DNMT3A* mutations at codon R882). Methylation abnormalities are likely to be involved in the causation of all the conditions. Given the vast number of methylation patterns that can exist on a single genome and the high likelihood that the DNA methyltransferase mutations will cause genome-wide methylation abnormalities, it might be extremely difficult to identify the specific methylation change that gives rise to a given biological effect in the DNA methyltransferase disorders. A recent report of characteristic methylation anomalies in *DNMT3*A^R882H/+^ or *DNMT3A* ^R882C/+^ cases of AML that are not present in *DNMT3A* ^+/+^ cases [52] highlights the issue: while the data strongly indicate that abnormal genomic methylation patterns are involved in the progression to AML, the methylation changes actually directly involved in leukemogenesis will be difficult to define.

## Conclusions

Many of the accepted views of the form, function, and dynamics of mammalian genomic methylation patterns were first formulated in the 1980s, when there was little information as to the true organization of the genome. A reappraisal in view of modern information leads to the conclusion that the dynamic demethylation and remethylation that occurs in early development affect largely unannotated sequences and inactive transposons, while imprinting control regions and potentially active transposons largely escape demethylation, and nearly all CpG-rich promoters are not methylated in any cell type. Genomic methylation patterns at sequences where methylation status might affect phenotype are much more static than previously believed, while methylation changes at sequences that are evolving at close to the neutral rate are unlikely to have biological consequences.

## Abbreviations

BAH domain: bromo-adjacent homology domain
CXXC domain: protein fold found in multiple proteins that bind to unmethylated CpG dinucleotides in DNA
DNMT: DNA (cytosine-5) methyltransferase
PRC1 and 2: polycomb repressive complexes 1 and 2. Involved in transcriptional repression, especially of genes involved in patterning the early embryo
RFTS domain: replication focus targeting sequence that recruits DNMT1 to replication foci during S phase
